# Curvature-dependent shear bond strength of different attachment materials for orthodontic lingual indirect bonding

**DOI:** 10.1038/s41598-021-96164-3

**Published:** 2021-08-16

**Authors:** Rebecca Jungbauer, Paul Al-Burghol, Martin Rosentritt, Christian Kirschneck, Peter Proff, Friedrich Paulsen, Christian M. Hammer

**Affiliations:** 1 Department of Orthodontics, University Medical Centre Regensburg, 93053 Regensburg, Germany; 2 Department of Prosthetic Dentistry, University Medical Centre Regensburg, 93053 Regensburg, Germany; 3 Institute of Functional and Clinical Anatomy, Friedrich Alexander University Erlangen-Nürnberg, 91054 Erlangen, Germany; 4 Department of Topographic Anatomy and Operative Surgery, Sechenov University, Moscow, Russia

**Keywords:** Medical research, Materials science

## Abstract

To evaluate the shear bond strength (SBS) of different attachment materials used for lingual bonding, the influence of artificial aging and the radii of curvature of the enamel surface on SBS, 192 third molars were photographed to determine the radius of curvature of the oral surface. After phosphoric acid etching a cylindrical test piece was bonded to the oral enamel using a mold that was filled with a chemically curing (Maximum Cure, Transbond IDB Premix) or a dual-curing (Nexus NX3, RelyX Unicem2) attachment material. SBS was tested after 24 h, 500 thermal cycles or 90 days at 37 °C with a universal testing machine. Computed tomography scans were performed to determine the bonded surface and calculate SBS. Values ranged from 8.3 to 20.9 MPa. RelyX Unicem2 showed the highest SBS values at baseline, 500 thermal cycles and after 90 days (*p* < 0.001). Ninety days of wet storage significantly reduced SBS of Maximum Cure (*p* = 0.028). The radius of curvature correlated positively with SBS (*r*_*s*_ = 0.204, *p* = 0.005). The SBS of all attachment materials was sufficient for clinical use, even after artificial aging. RelyX Unicem2 showed almost twice as high SBS values as the other attachment materials.

## Introduction

Fixed appliances are a common and reliable treatment option in modern orthodontics. Traditionally, orthodontists have been bonding brackets on the labial, visible part of the teeth to correct malocclusions. In recent years, the number of adults seeking orthodontic treatment has been constantly increasing^1^. Due to aesthetic reasons most adults desire an invisible treatment appliance^2^. In 1979 Fujita was the first to describe the insertion of a multi-bracket-appliance on the inner, lingual/palatal side of the teeth^3^. Since then, this kind of treatment option has been refined substantially and is gaining more and more popularity^4,5^. Because of the high risk of bonding errors due to limited visual access, lingual brackets are usually indirectly bonded with the help of a bonding transfer tray after the bracket position has been carefully planned in a laboratory or via a digital process^4,6^. Consequently, rebonding after accidental debonding during treatment requires an increased effort compared to the labial technique and bracket loss needs to be kept to a minimum^7^. The bonding between enamel and the bracket, respectively the attachment material (AM), is required to be stable during treatment without causing any damage to the enamel. In the literature shear bond strength values between 5 and 10 MPa are recommended^8,9^.

The shear bond strength (SBS) of different AMs has been tested in many studies^10–14^. However, most studies investigated light-curing AMs used for direct bonding. In general, there are few studies investigating AMs used in lingual bonding, respectively indirect bonding techniques. The aim of this study was to compare the SBS between human enamel and the four most commonly used AMs recommended by the leading manufacturers of customized lingual appliances^15,16^. These are: (1) Maximum Cure® (Reliance Orthodontic Products, Inc., Itasca, USA), (2) Transbond™ IDB Pre-Mix (3 M, Monrovia, USA), (3) NX3 Nexus™ (Kerr Corporation, Orange, USA) and (4) RelyX™ Unicem2 Automix (3 M, Monrovia, USA). It was hypothesized that (a) there is no difference in SBS between the different AMs, (b) different aging methods do not have any influence on SBS, (c) the way of curing (chemical or dual cure) does not influence the SBS and (d) there is no correlation between SBS and the radii of curvature. These hypotheses were tested by laboratory SBS measurements involving extracted human third molars and a biomaterial testing device.

## Materials and methods

This study was performed on the basis of the DIN 13990:2017-04^17^ standard. However, not only rather flat surfaces (radius of curvature ≥ 12.5 mm) were used as bonding surface.

### Sample preparation

One hundred and ninety-two human third molars (96 upper, 96 lower), which had been extracted for medical reasons from patients aged between 12 and 40 years, were collected for this study. All crowns were free of restorations, caries, scratches or fracture lines. Approval for the collection and use of human teeth extracted for medical reasons was given by the ethics committee of the University of Regensburg, Germany (Approval No. 12-170-0150). All methods were carried out in accordance with relevant guidelines and regulations. Informed consent was obtained from all participants or, if participants ﻿were under 18, from a parent and/or legal guardian.

**Figure 1:**
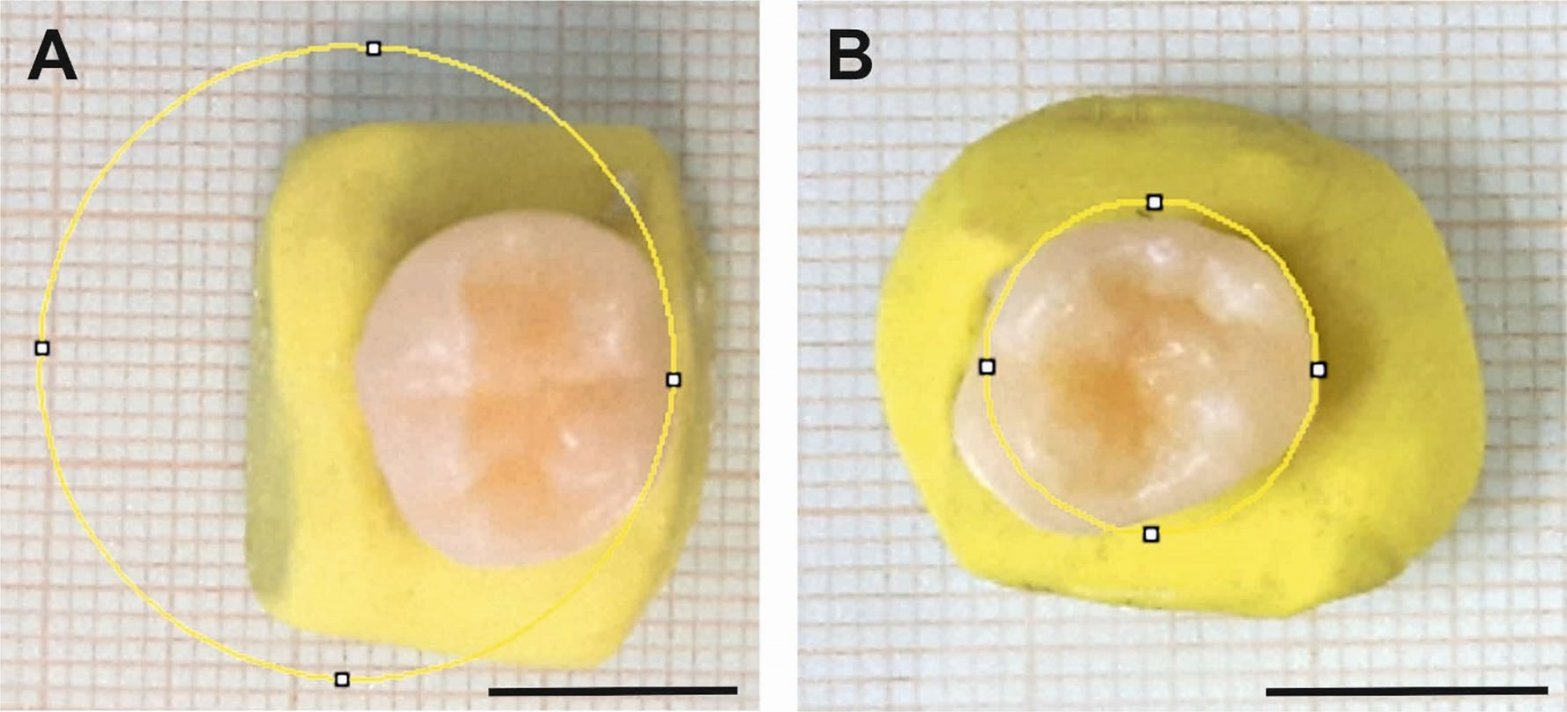
Determination of the lingual/palatal radii of curvature. The photographs show the occlusal side of two representative tooth samples. The lingual/palatal surface is oriented to the right. A horizontal circle (yellow) was digitally fitted to the labial/palatal side of each sample. The radius of the best fit was recorded for each specimen and considered the respective radius of curvature. (**A**) Large radius of curvature. (**B**) Small radius of curvature. Scale bars: 1 cm.

After extraction, the teeth were first stored in 0.5% chloramine-T-solution for 1 week at room temperature and afterwards in distilled water at 4 °C. The horizontal radius of curvature of every tooth sample was assessed before bonding. For that purpose, the occlusal aspect of the teeth was photographed together with a scale. By use of the open-source Software Fiji^18^ the horizontal circle that fitted best to the lingual/palatal side (where the AM was bonded afterwards) of the teeth was chosen (Fig. 1). After calibration, the radius of the best fitting circle was recorded as the radius of curvature.

Prior to the SBS measurements all third molars were fixed using dental technician wax with the lingual/palatal surface oriented upwards and parallel to the base in a custom-made device consisting of polytetrafluoroethylene, which had been constructed according to DIN 13990:2017-04^17^ requirements. The teeth were then embedded in fast-setting resin (Technovit 4000, Hereaus Kulzer, Werheim, Germany) within this device. During polymerization of the resin, specimens were stored in cold water for five minutes to prevent overheating as recommended by the DIN 13990:2017-04^17^. After removing the specimens from the device, sharp edges were smoothened with a dental milling machine. Until bonding, all specimens were stored in distilled water at room temperature.

Before bonding, the lingual/palatal surfaces of the teeth were cleaned with a polishing brush (Busch & Co, Engelskirchen, Germany) and a pumice/water mixture (40 g:50 g) for 3 s moving from the mesial to the distal side and 3 s from the occlusal to the gingival side at a speed of 3000 rounds per minute. The AM was bonded with the help of a silicone mold with a cylindrical cavity with a diameter and height of 3 mm each (TFC Silikon Kautschuk Typ 14 transluzent, Troll Factory, Riede, Germany; shore hardness 42A), which was manufactured according to DIN 13990:2017-04^17^. Before bonding, the specimens were divided into upper and lower third molars and randomly assigned to one of the testing groups described below (n = 16/group), each group containing the same number of upper and lower teeth.

**Figure 2:**
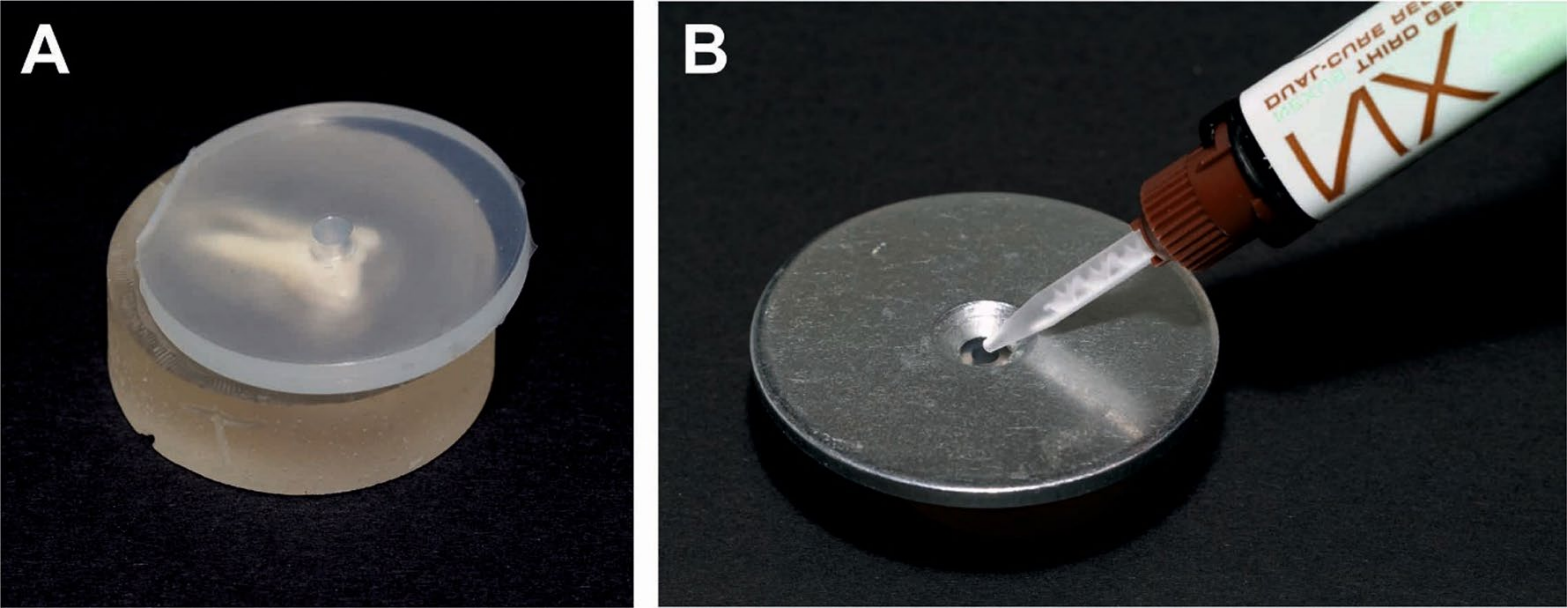
Illustration of attachment material application. (**A**)**:** Custom-made silicone mold on the lingual/palatal side of the crown of a third molar embedded in Technovit resin. (**B**) Placement of a standardized shim (diameter: 51 mm, weight: 46. 55 g) on top of the silicone mold. Application of Nexus NX3 with a mixing syringe is shown as an example.

The enamel was pretreated with 35% phosphoric acid (iBond, Hereaus Kulzer, Werheim, Germany) for 30 s, rinsed with water and air-dried with oil-free air until a frosty appearance was visible. The silicone mold was placed on the lingual/palatal side of the teeth (Fig. 2A) and held in place by the weight of a shim with the diameter of 53 mm (Fig. 2B). The chemical curing AMs, Maximum Cure (MC, groups 1, 5, 9) and Transbond IDB Premix (IDB, groups 2, 6, 10), were mixed in a dappen dish by use of a microbrush and filled in the silicon mold. During polymerization, the mold was kept in place for 5 min and was removed afterwards. After removal of the mold, a cylindrical stub of polymerized AM (adhesive cylinder) was protruding orthogonally from the lingual/palatal tooth surface. The dual curing AMs, Nexus NX3 (NX, groups 3, 7, 11) and RelyX Unicem2 (RXU, groups 4, 8, 12), were applied by syringes with mixing tips (Fig. 2B).

The first fraction of material coming out of the mixing tip was always discarded. The second fraction of the AM was then poured into the silicone mold and cured by light for 20 s with an intensity of 1.492 mW/cm^2^ using a MiniLED (ACTEON, Düsseldorf, Germany). Directly after bonding, all specimens were put in interim storage in distilled water at room temperature. Then, groups 1–4 were stored in distilled water at 37 °C for 24 h, groups 5–8 underwent 500 thermal cycles (5/55 °C, dwelling time: 2 min; eGo-Kälteysteme GmbH, Regensburg, Germany) and groups 9–12 were stored in distilled water at 37 °C for 90 days. The composition of the attachment materials is presented in Table 1.

**Table 1:** Manufacturer and composition of the attachment materials used in this study.

Materials	Manufacturer	Composition
Maximum Cure (MC)	Reliance Orthodontic Products	Part A (Bis-GMA^a^, hydrofluoric acid 7%, stabilizers)
		Part B (bis-GMA^a^, dibenzoyl peroxide, stabilizers)
Transbond IDB Premix (IDB)	3M	Part A (Bis-GMA^a^, TEGDMA^b^, benzoyl peroxide, stabilizers)
		Part B (Bis-GMA^a^, TEGDMA^b^, stabilizers)
Nexus 3 (NX)	Kerr Corporation	TEGDMA^b^, Bis-GMA^a^, fluoroaluminosilicate glass, activators, stabilizers, radiopaque agent
RelyX Unicem 2 Automix (RXU)	3M	TEGDMA^b^, methacrylate monomers containing phosphoric acid groups, sodium *p*-toluenesulfinate, pigments, silanated fillers, activators, radiopaque agent

^a^Bisphenol *a*-glycidyl methacrylate (Bis-GMA).

^b^Triethylene glycol dimethacrylate (TEGDMA).

### Shear bond strength measurement

**Figure 3:**
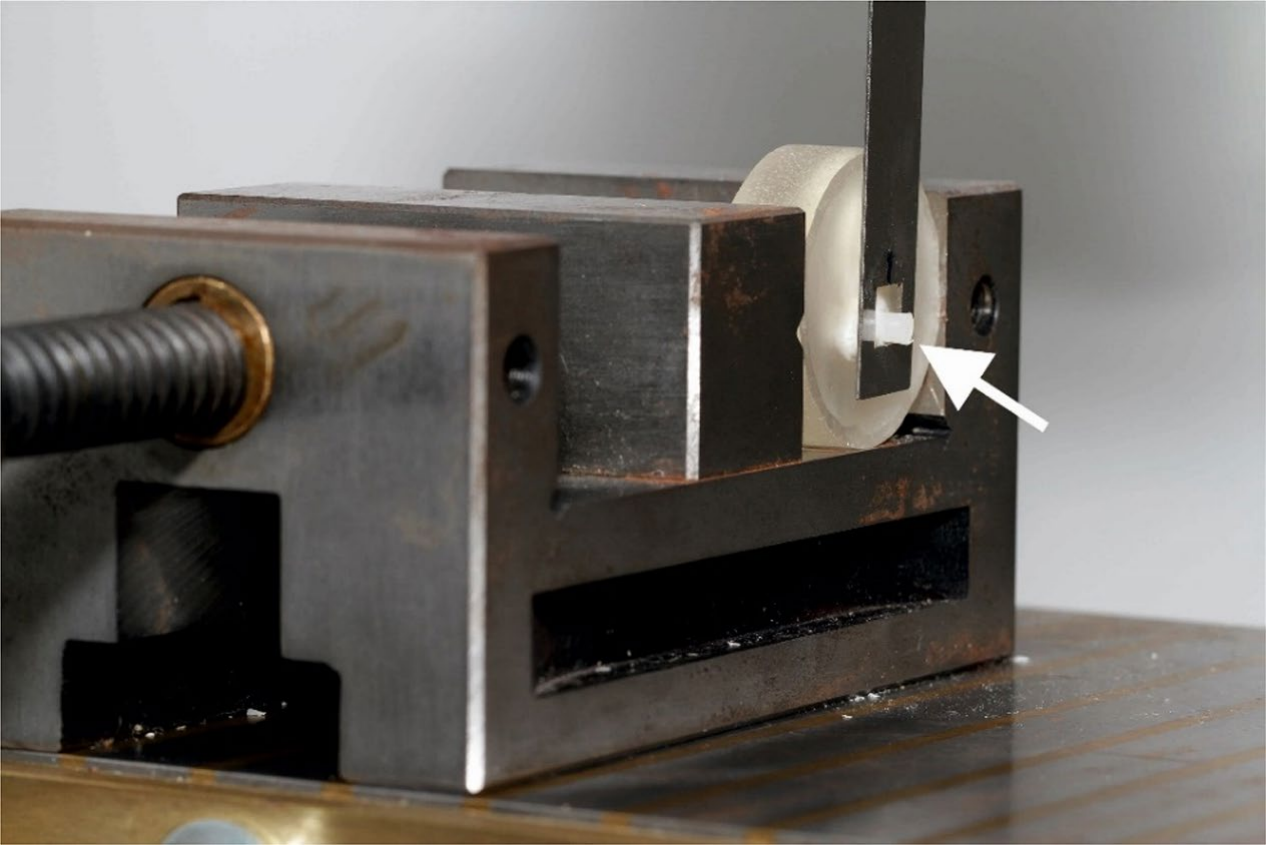
Experimental setup of the SBS measurements. The tooth sample is fastened in a fixed metal vise with the occlusal surface of the crown facing downwards. The adhesive cylinder protruding from the lingual/palatal surface is reaching through the rectangular hole in the metal blade (arrow). The blade is connected to a force sensor and moved in occlusal-gingival direction (upwards). This way, the adhesive cylinder is sheared off the tooth and the developing forces are recorded, simultaneously.

Prior to the shear bond strength measurements, the specimens were stored in distilled water at room temperature for 1 h. Shear bond strength was measured with the universal testing machine Instron 5965 (Instron Deutschland GmbH, Pfungstadt, Germany). The samples were fixed in a magnetically lockable device at the base of the testing machine. A metal blade containing a rectangular hole measuring 6 mm × 6 mm, which was used to shear off the adhesive cylinders from the teeth, was fabricated according to DIN 13990:2017-04^17^ and fastened to the movable part of the testing device. The tooth surface carrying the polymerized AM was carefully positioned parallel to the blade with the AM adhesive cylinder reaching through the rectangular hole (Fig. 3). The force was applied to the interface between enamel and AM by moving the blade in an occlusal-gingival direction with a crosshead speed of 1 mm/min until failure. The maximum force was recorded in Newton.

**Figure 4:**
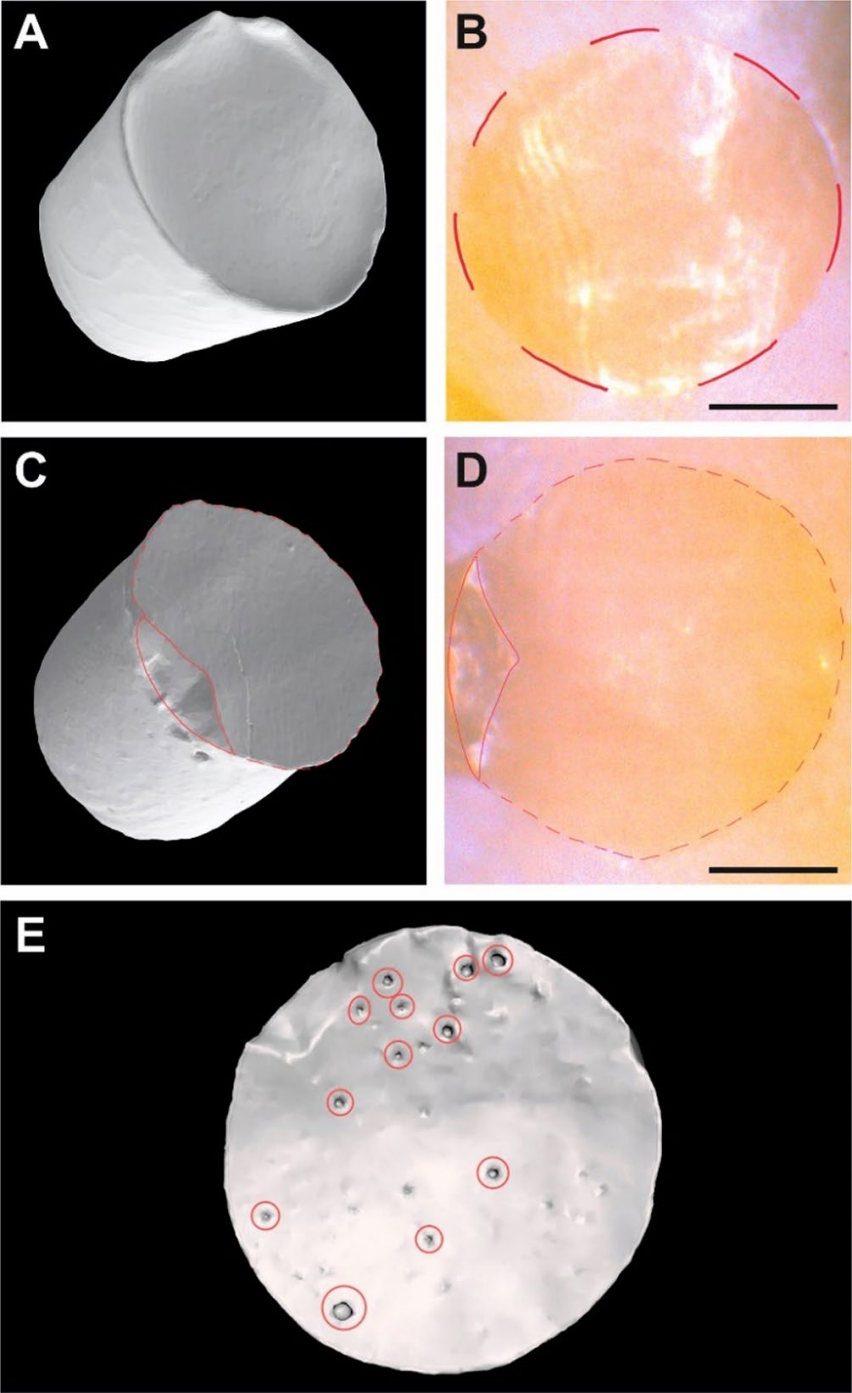
Determination of the effective bonding area. (**A**) Three-dimensionally reconstructed CT scan of an intact adhesive cylinder. (**B**) Microscopical image of the corresponding bonding area on the enamel. (**A**,**B**) No virtual reconstruction of the bonding area was necessary for the calculation of SBS. (**C**) Three-dimensionally reconstructed CT scan of a damaged adhesive cylinder. The missing area is marked with a red line. (**D**) Microscopical image of the corresponding bonding area on the enamel. The part that is missing in C is still attached to the enamel and marked with a red line. (**C**,**D**) Virtual reconstruction of the effective bonding area was necessary for the calculation of SBS. (**E**) Detection of gas bubbles (red circled) reducing the bonding area. The areas occupied by gas bubbles were determined on the CT scan and then subtracted from the total area. This way, the effective bonding area was calculated. Scale bars: 1 mm.

All specimens were then photographed under a microscope (Bresser, Rhede, Germany) with 10 × magnification and screened for cracks. Due to the varying degree of enamel curvature, the bases of the cylinders that were bonded with the flexible silicone mold, did not have a uniform diameter of 3 mm. To accurately determine the area bonded to the enamel surface all adhesive cylinders were collected after the force measurement and digitized with an industrial computer tomograph (Zeiss Metrotom 800, Zeiss, Oberkochen, Germany). The resulting dataset was converted into the STL format using the software VGStudioMax 3.2 (volumegraphics, Heidelberg, Germany). To calculate the exact bonding area, every cylinder was analyzed using the software Meshmixer (Autodesk, Mill Valley, USA). First, the area of the adhesive cylinder surface that had been bonded to the enamel (Fig. 4A,C) was compared to the respective microscope image displaying the counterpart area of bonding on the corresponding tooth (Fig. 4B,D). On this image the different shades on the surfaces of the etched teeth were distinguished to identify the outline of the surface that the adhesive cylinder was bonded to. This way, it was possible to determine, if a reconstruction of the adhesive cylinder surface as a result of chipping during the SBS test was necessary. In these cases, the marginal ridge of the area of the adhesive cylinder was thus virtually reconstructed (Fig. 4C,D) and the area filled before determination of the surface area. In case of bubbles present on the surface of the adhesive cylinder, the bubble area on the bonding surface was also calculated (Fig. 4E) and subtracted from the entire bonding surface. Thus, the area of the bubbles was not included in the effective bonding area used for SBS calculation. The bonding strength was calculated for every specimen with the formula:

$$R\, (\mathrm{N}/{\mathrm{mm}}^{2}) =\frac{F (\mathrm{N})}{A ({\mathrm{mm}}^{2})}$$

here *R* represents the SBS in Megapascal (MPa = N/mm^2^), *F* constitutes the maximum force in Newton (N) before failure, and *A* is the effective bonding area in square millimeters (mm^2^).

The adhesive remnant index (ARI) was evaluated as follows: 0 = no remaining AM on the bonded enamel surface, 1 = AM remaining on less than 50% of the bonded enamel surface, 2 = AM remaining on more than 50% of the bonded enamel surface, 3 = AM remaining on 100% of the bonded enamel surface^19^. A random sample from each AM group was sputter-coated with a 20 nm layer of gold using the Leica EM ACE200 system (Leica Mikrosysteme GmbH, Vienna, Austria) and viewed with a JEOL scanning electron microscope at 30 × and 250 × magnification (JSM-IT 300LV, JEOL Germany GmbH, Eching, Germany).

### Reliability of measurements

Twenty specimens were randomly selected, and the assessment of ARI was performed a second time by the same investigator and additionally by a second experienced investigator. To calculate intra- and interrater reliability the intraclass correlation coefficient (ICC; two-way mixed, absolute agreement) selected was used.

### Statistical analysis

Data were analyzed using SPSS version 24.0 (IBM Armonk, NY, USA). For descriptive statistics of the SBS data, medians and interquartile ranges (IQR) as well as means and standard deviations (SD) were calculated for every treatment group. Normal distribution was tested with a *Shapiro–Wilk*-Test and visual assessment of histograms. Non-parametric *Kruskal–Wallis*-tests followed by post hoc *Dunn–Bonferroni*-tests and *Mann–Whitney-U*-tests using the *Bonferroni* correction were applied to check, if the differences between groups were statistically significant. To test the correlation between SBS and the radii of curvature *Spearman’s* rank correlation coefficient was calculated. The Chi^2^ test was applied to evaluate the ARI. *p* values of *p* ≤ 0.05 were interpreted as statistically significant.

### Ethical approval

Approval for the collection and use of human teeth extracted for medical reasons was given by the ethics committee of the University of Regensburg, Germany (Approval No. 12-170-0150).

## Results

Overall, SBS between enamel and RXU showed the highest values. The difference was significant in comparison to all the other adhesives after 500 thermal cycles (RXU: 18.1 MPa; MC: 10.4 MPa, *p* = 0.009; NX: 10.1 MPa, *p* = 0.001; IDB: 9.6 MPa, *p* = 0.002) and 90 days of wet storage (RXU: 19.8 MPa; IDB: 10.7 MPa, *p* = 0.002; NX: 10.6 MPa, *p* = 0.003; MC: 8.3 MPa, *p* < 0.001). At 24 h after bonding RXU, SBS values (20.9 MPa) were significantly higher than after bonding with IDB (12.9 MPa, *p* = 0.017) and NX (9.4 MPa, *p* = 0.013). SBS values of MC were lower (13.7 MPa) but without statistical significance. MC was the only AM that showed a significant decrease in SBS values after 90 days of wet storage compared to the baseline measurement after 24 h (*p* = 0.028). The detailed results are presented in Table 2. The mean radius of curvature of all samples was 9.5 mm (SD: 5.1 mm; Min: 3.0 mm; Max: 25.9 mm). Higher SBS values correlated significantly with larger radii of curvature (*r*_*s*_ = 0.204, *p* = 0.005). In general, dual curing adhesives showed significantly higher mean SBS compared to chemical curing adhesives (13.2 MPa vs. 10.3 MPa, *p* < 0.001).

**Table 2:** Mean and median shear bond strengths of all experimental groups in MPa.

Adhesive	Storage/aging					
	24 h		500 cycles		90 days	
	Median (IQR)	Mean ± SD	Median (IQR)	Mean ± SD	Median (IQR)	Mean ± SD
MC	13.7 (14.1)^ABa^	14.5 ± 6.8	10.4 (5.8)^Aab^	11.5 ± 4.0	8.3 (4.3)^Ab^	8.7 ± 3.3
IDB	12.9 (9.0)^Ba^	11.9 ± 5.6	9.6 (5.8)^Aa^	10.9 ± 3.6	10.7 (5.0)^Aa^	11.1 ± 4.2
NX	9.4 (7.8)^Ba^	12.1 ± 7.3	10.1 (4.6)^Aa^	10.4 ± 3.6	10.6 (3.3)^Aa^	11.4 ± 4.7
RXU	20.9 (8.6)^Aa^	19.5 ± 5.1	18.1 (7.5)^Ba^	17.8 ± 5.4	19.8 (9.0)^Ba^	19.5 ± 5.4

Different attachment materials are represented by the rows. Different storage/aging methods are represented by the columns. Statistical significance of differences (*p* ≤ 0.05) is indicated by superscript letters. Values that do not differ significantly share the same letter. Values that differ significantly are marked with different letters. Different uppercase letters indicate significant differences (*p* ≤ 0.05) in the columns (between attachment materials). Different lowercase letters indicate significant differences in the rows (between storage/aging methods). *MC* maximum cure, *IDB* transbond IDB Premix, *NX* Nexus NX3, *RXU* RelyX Unicem2.

The ARI was related to the different AMs utilized (Chi^2^ < 0.001). In both chemical curing groups (MC, IDB) an ARI score of 1 was detected most frequently (72.9% and 70.8%, respectively). In the NX group the most frequently encountered ARI score was 0 (56.3%), followed by 1 (41.7%). In the RXU group, an ARI of 1 was encountered most frequently (64.6%), followed by an ARI of 2 (12.5%) and 3 (4.2%). The detailed results are presented in Table 3.

**Table 3:** Distribution of ARI.

	ARI				Total
	0	1	2	3	
**Attachment material**					
MC	13 (27.1%)	35 (72.9%)	0 (0%)	0 (0%)	48 (100%/25%)
IDB	11 (22.9%)	34 (70.8%)	3 (6.3%)	0 (0%)	48 (100%/25%)
NX	27 (56.3%)	20 (41.7%)	1 (2.1%)	0 (0%)	48 (100%/25%)
RXU	9 (18.8%)	31 (64.6%)	6 (12.5%)	2 (4.2%)	48 (100%/25%)
Total	60 (31.3%)	120 (62.5%)	10 (5.2%)	2 (1.0%)	192 (100%/100%)

Number and percentage of samples with the respective ARI (0–3). Data are presented for every attachment material separately and for all samples taken together. *MC* maximum cure, *IDB* Transbond IDB Premix, *NX* Nexus NX3, *RXU* RelyX Unicem2.

**Figure 5:**
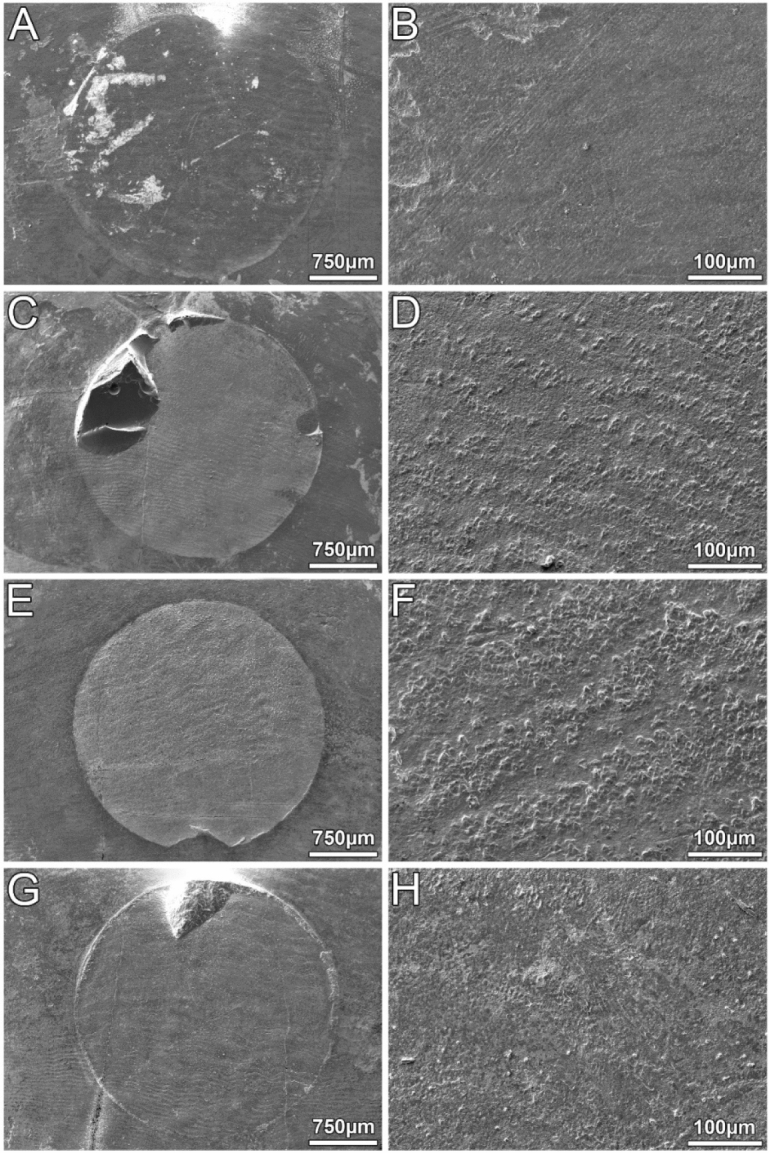
Scanning electron micrographs of the bonding areas on the lingual/palatal sides of the third molar crowns after SBS measurement. Left column: general views at 30-fold magnification. Circular bonding areas are discernible. Right column: detailed views of the central bonding areas shown in the respective row of the left column (250-fold magnification). No obvious damage to the enamel observable. Wavelike patterns presumably representing Hunter-Schreger lines are discernible in all specimens displayed. (**A**,**B**) Maximum Cure. (**C**,**D**) Transbond IDB Premix. Pronounced remnant of the adhesive cylinder in the upper left quadrant. (**E**,**F)** Nexus NX3. (**G**,**H**) RelyX Unicem2. Small remnant of the adhesive cylinder at the twelve o’ clock position.

Scanning electron microscopic images of the bonding surface on the lingual/palatal side of the third molar crowns after the SBS measurement are displayed in Fig. 5 for all experimental AM groups. The circular bonding area was easily discernible in all specimens. The samples representing the IDB and the RXU groups show remnants of the adhesive cylinder. No fractures, fissures or other forms of damage were found on the enamel. Instead, the typical wavelike pattern, possibly representing Hunter-Schreger bands, was observed on the enamel surface. These patterns were not confined to the bonding area but were present all over the lingual/palatal sides of the molar crowns.

### Reliability of measurements

The intra- as well as the interrater reliability were both excellent with an ICC of 0.969.

## Discussion

The aim of this in vitro investigation was to compare the shear bond strength (SBS) of four different orthodontic attachment materials (AMs) that are commonly used for indirect bonding of lingual appliances. Moreover, the influence of different aging methods and the curvature of the enamel surface on SBS was examined.

The first hypothesis (“There is no difference in SBS between the different AMs.”) had to be rejected, as the results of this study clearly show significantly higher SBS values between human enamel and RXU in comparison to the other AMs. RXU is a dual cure self-adhesive resin cement normally used for prosthodontic purposes, such as the cementation of restorations and posts^20,21^. Two studies reported that, in comparison to Transbond XT (3 M, Monrovia, USA), RXU did not show sufficient SBS for bonding labial brackets^22,23^. In those studies, the enamel surface was not etched with 35% phosphoric acid before bonding the brackets with RXU. The functional acidic monomers that are contained in self-adhesive resin cements such as RXU are considered to be weaker in comparison to selective enamel etching using phosphoric acid, which results in a lower bonding strength to enamel. As a consequence, additional enamel etching is recommended, for example, for cementing veneers to enamel^20^. In the present study, enamel etching was performed for 30 s before bonding the adhesive cylinders, resulting in higher SBS values for RXU.

In accordance with our study, Scribante et al. found significantly higher maximum force values until debonding of RXU in comparison to MC and IDB for customized gold-alloy base brackets that were indirectly bonded^24^. Enamel surfaces of premolars were pretreated with sandblasting (27 μm aluminum oxide) and 37% phosphoric acid (30 s). Remarkably, they even described a significantly higher rate of enamel fractures after debonding for RXU^24^. The maximum force values after bonding with either MC or IDB were comparable and did not differ significantly^24^, which is also in line with the present results. Cal-Neto et al. found sufficiently high SBS values of 13.17 ± 4.33 MPa for a metal lingual bracket customized with a resin base bonded indirectly to human enamel with MC^25^. The use of NX3 for the bonding of brackets has not been evaluated previously. The results of the present investigation clearly indicate similar SBS values for NX3 in comparison to MC and IDB.

MC and IDB are both chemical curing attachment materials, NX3 and RXU are dual curing. Moreover, RXU is also self-adhesive. Although the statistical analysis indicates significantly higher SBS values for dual curing attachment material, this result has to be viewed with caution. A closer look on the data reveals that this is mainly caused by the much higher SBS values found in the RXU groups. Especially after 90 days of storage the RXU-related SBS values are twice as high when compared to the other AMs. Therefore, the third hypothesis (“The way of curing (chemical or dual cure) does not influence the SBS.”) can be largely accepted. The functional acidic monomers contained in the self-adhesive RXU enhance surface demineralization of the enamel^20^. This fact might contribute to the much higher SBS values in comparison to MC, IDB and NX3.

Currently, there are only few studies investigating SBS in the context of the lingual bonding technique. Sha et al. investigated the differences in SBS of different types of lingual and labial brackets, preadjusted and customized, all of them bonded with RXU, and found similar SBS values for the two types of customized lingual brackets used^6^. The highest value of SBS was found in the labial customized group with a resin as customized base^6^. Sung et al. reported a significantly higher SBS for limited resin custom bases in comparison to extended resin and gold-alloy custom bases^26^. SBS values in the present study were generally higher than in the above-mentioned studies. Considering the experimental setting for SBS testing, the force application point will be different as soon as the shapes of the brackets and their bases differ, especially their thickness. The curvature of most customized lingual brackets makes a reproducible force application even more difficult within the available experimental setups. Consequently, different ratios of direct force and moment of force application have an influence on the outcome. Therefore, it was our approach, in accordance with the DIN 13990:2017-04^17^, to have a closer look at the performance of the bonding quality only between lingual/palatal enamel and the different AMs. The bonding strength of the interface between different types of brackets and AMs needs to be considered separately, as there are too many factors that have an influence on SBS. Additional customized resin interfaces or different metal surfaces and their possible pretreatments are good examples for that. As a result, the SBS values presented here can hardly be compared to those of other studies. Nevertheless, SBS values of at least 5 MPa are generally considered to be sufficient for multi-bracket-treatment^8,9^. Restricted to the interface between enamel and AM, all the tested materials showed sufficiently high SBS values.

In the present study, thermal cycling did not have a significant influence on SBS. Nevertheless, the second hypothesis (“Different aging methods do not have an influence on SBS.”) had to be partly rejected, as SBS values of MC were significantly lower after 90 days of wet storage at 37 °C. In general, in-vitro investigation of the long-term performance of the tested materials is also of importance for the assessment of their stability and clinical applicability^27^. In contrast to materials utilized in restorative dentistry, the requirements for a reliable bonding in fixed orthodontic therapy is time-limited, as the treatment lasts on average about 2 years^28^. Therefore, a smaller number of thermal cycles was considered to be sufficient. In accordance with the DIN 13990:2017-04, all specimens in the thermal cycling group underwent 500 cycles^17^. Thermal cycling imitates daily intraoral temperature fluctuations that occur when patients eat/drink cold and hot food/beverages. This results in inner stress in the AM and over time leads to degradation. Inner stress can be a result of differences in thermal expansion and shrinking between filler particles and the resin^29^. Furthermore, microleakage or even debonding can be caused by differential thermal expansion of the AM and the tooth structure^29^. Apart from thermal cycling, the second most applied method for artificial aging of resins is wet storage^27^. Wet environment can lead to degradation of restorations^29^ and therefore also have an influence on the AMs that are used to bond brackets. In the present investigation, MC showed significantly lower SBS values after 90 days of wet storage at 37 °C, which might be caused by weakening of the attachment material due to degradation of the fillers, softening, and hydrolysis^29^. Compared to IDB and NX3, the SBS values were lower but not significantly. RXU still showed very high SBS values after wet storage aging. When the functional acidic monomers have reacted, RXU becomes more hydrophobic and the tendency to absorb water, expand, and hydrolytically degrade is minimized^20^. In summary, thermal cycling and/or wet storage are essential parts of testing orthodontic attachment materials. However, no previous study investigating SBS of attachment materials for lingual brackets has taken this aspect into account.

The fourth hypothesis (“There is no correlation between SBS and the radii of curvature.”) was rejected as well. The results of the present study showed an influence of the radii of curvature on SBS in terms of smaller radii of curvature (rather curved surfaces) correlating with lower SBS values and larger radii of curvature (rather flat surfaces) with higher SBS values. According to the DIN 13990:2017-04, it is recommended to use rather flat (radius of curvature ≥ 12.5 mm) specimens to test SBS. Since lingual brackets are bonded to the lingual/palatal surfaces, that vary considerably in terms of their curvature, it seems prudent not to use flat surfaces in these special cases. As the results of the present study suggest, it seems to be important to determine and consider the radii of curvature when brackets are bonded to curved surfaces for SBS testing. To date, this aspect has not been considered by any other investigation dealing with orthodontic SBS testing. However, this aspect may be of clinical relevance as the clinician might consider the application of an attachment material with higher SBS values when bonding to very curved surfaces. This might be especially crucial for the rebonding of brackets after accidental debonding during active treatment.

After the adhesive cylinder had been sheared off, the ARI was determined. An ARI of 0 means that no AM was left on the enamel surface, which results in less clinical chair time to remove the adhesive, but at the cost of a higher risk of fracture^26^. With exception of NX3 an ARI of 1 was most common, which is in accordance with Scribante et al.^24^. However, as this is the first study on the SBS only between enamel and AM, the present ARI results can hardly be compared to those from other studies and the clinical relevance is therefore limited. Nevertheless, the results show that RXU was the only AM the bonding was strong enough to cause two of the resin cylinders to crack within themselves, leading to the whole enamel surface still being covered with RXU. This is in line with the much higher SBS values of RXU.

SBS testing is often criticized for several weak points. Due to the large number of different experimental setups and testing procedures, the results are very often difficult to compare. Therefore, it is essential to adhere to standardized protocols as far as possible if a specific research question is to be answered validly. In general, shear tests show several limitations compared to tensile tests, especially micro-tensile bond strength, such as a higher stress concentration e.g. due to deviations of the moduli of the materials involved^30–32^. This may well influence results and fracture patterns. However, shear tests are less complex, easier to perform, less costly, and take into account the surface configuration of the substances being tested. Due to this and the near-clinical loading situation, shear tests with a detailed description of the specimen setup and test configuration are good to use as comparative measurements. Furthermore, the long-term aspect regarding artificial aging should always be considered. This way, SBS testing provides a good possibility to compare the performance of different materials and to use these results as a basis for clinical trials. In this study, only the bonding between enamel and AM was investigated. Further research is required to consider all the other aspects, such as different bracket shapes and materials or customized resin pads. For this purpose, a standardized procedure for shearing customized brackets off will be crucial.

## Conclusions

Within the limitations of this study, the following conclusions can be drawn:

- All investigated AMs showed sufficiently high SBS values concerning the interface between enamel and attachment material.
- SBS between RXU and enamel was significantly higher than between enamel and the other AMs tested.
- After 90 days of wet storage SBS values of MC were significantly lower than after 24 h.
- The curvature of the bonded surface correlates negatively with SBS.

## Data Availability

The datasets generated and/or analyzed during the current study are available from the corresponding author on reasonable request.
